# Sex-specific alterations in CRF and dopaminergic markers in the lateral septum and mesolimbic system following chronic high-fat diet exposure in rats

**DOI:** 10.3389/fphar.2026.1781720

**Published:** 2026-04-30

**Authors:** Rossy Olivares-Barraza, José L. Marcos, Jorge Escobar-Luna, María José Covarrubias, Georgina M. Renard, Patricio Iturriaga-Vásquez, Katia Gysling, Javier A. Bravo, Marco Fuenzalida, Ramón Sotomayor-Zárate

**Affiliations:** 1 Centro de Neurobiología y Fisiopatología Integrativa (CENFI), Instituto de Fisiología, Facultad de Ciencias, Universidad de Valparaíso, Valparaíso, Chile; 2 Programa de Doctorado en Ciencias Mención Neurociencia, Universidad de Valparaíso, Valparaíso, Chile; 3 Instituto de Biología, Facultad de Ciencias, Pontificia Universidad Católica de Valparaíso, Valparaíso, Chile; 4 Programa de Doctorado en Ciencias e Ingeniería para la Salud, Universidad de Valparaíso, Valparaíso, Chile; 5 Departamento de Ciencias Veterinarias, Facultad de Ciencias de la Vida, Universidad Viña del Mar, Viña del Mar, Chile; 6 Centro de Investigación Biomédica y Aplicada (CIBAP), Escuela de Medicina, Facultad de Ciencias Médicas, Universidad de Santiago de Chile, Santiago, Chile; 7 Departamento de Ciencias Químicas y Recursos Naturales, Facultad de Ingeniería y Ciencias, Universidad de la Frontera, Temuco, Chile; 8 Grupo de NeuroGastroBioquímica, Instituto de Química, Facultad de Ciencias, Pontificia Universidad Católica de Valparaíso, Valparaíso, Chile; 9 Millennium Nucleus of Neuroepigenetics and Plasticity (EpiNeuro), Santiago, Chile

**Keywords:** CRF system, dopamine, high-fat diet, lateral septum, obesity

## Abstract

**Background:**

Obesity is a global health concern closely associated with sedentary lifestyles and the consumption of hypercaloric, hyperpalatable diets. While these factors contribute to excessive food intake and weight gain, the long-term impact of obesogenic diets on neural circuits regulating energy balance remains insufficiently understood. Previous research has identified the lateral septum (LS) as a critical structure involved in stress responses and addiction, with corticotropin-releasing factor (CRF) and dopaminergic signalling playing key roles. However, the influence of chronic high fat diet (HFD) exposure on LS function and its contribution to feeding regulation remains poorly characterized.

**Methods and Results:**

Male and female *Sprague–Dawley* rats were fed either a high fat diet (HFD) or standard chow from postnatal day 21–62. Chronic HFD exposure induced anxiolytic-like behavior and increased CRF in the LS of males. In females, HFD elevated dopamine D_1_ and D_2_ receptor expression, as well as CRF receptor type 2 (CRF_2_), compared to controls. Moreover, dopamine metabolite levels were elevated in mesolimbic regions in both sexes following HFD exposure.

**Conclusion:**

These results indicate that the LS undergoes sex-specific molecular and cellular alterations in response to prolonged HFD consumption. The observed alterations in dopamine and CRF signalling suggest that the LS may mediate diet-induced dysregulation of both homeostatic and hedonic aspects of feeding. These findings provide novel insights into the neural mechanisms by which chronic exposure to an obesogenic diet may promote overeating and obesity.

## 1 Introduction

The World Health Organization (WHO, 2024) has declared obesity a pandemic, characterized by an excessive accumulation of body fat and represents a major risk factor for chronic non-communicable diseases associated with an elevated body mass index (BMI: kg/m^2^) (Skinner et al., 2018). World Obesity Federation (WOF) has estimated a global prevalence of 1.53 billion people who will have a high BMI in 2035 (WOF, 2025). Obesity is considered a multifactorial condition influenced by factors such as unhealthy dietary habits, sedentary lifestyles, socioeconomic status, and, less frequently, genetic disorders (McArdle et al., 2013). Despite the contribution of these variables, the consumption of hypercaloric and hyperpalatable foods is often identified as a predominant factor in the development of obesity (Ifland et al., 2009), affecting the overall functioning of the body, including brain function.

The brain integrates neuronal signals related to energy metabolism, regulating homeostatic food intake in response to hunger and satiety cues (Volkow et al., 2017). In addition, dietary components such as sugars and fats activate brain reward circuits, promoting hedonic eating even in the absence of hunger (Berthoud and Munzberg, 2011; Egecioglu et al., 2011; Lee and Dixon, 2017). Peripheral hormonal signals, like ghrelin, insulin, and leptin, facilitate the homeostatic regulation of food intake, these signals regulate the activity of orexigenic and anorexigenic hypothalamic neurons (Marcos et al., 2023). Regard to hypothalamic network, the paraventricular nucleus (PVN), an anorexigenic hypothalamic area, is inhibited by GABAergic neurons of the lateral hypothalamus (LH), triggering the hunger sensation (Olszewski et al., 2003). In addition, lateral septum (LS), a GABAergic nucleus (Swanson and Cowan, 1979; Skibicka et al., 2012), receives hippocampal glutamatergic afferents that activate LS GABAergic neurons (Carnes et al., 1990), while the main LS GABAergic efferents go to LH (Jakab and Leranth, 1995), inhibiting its activation. Recently, it was shown that the hippocampus (Hipp)-LS-LH pathway controls food intake (Sweeney and Yang, 2015; 2016).

On the other hand, hedonic control of feeding is related to rewarding and emotional factors that regulate food intake (Kenny, 2011; Lee and Dixon, 2017) and it is formed by midbrain dopaminergic neurons of the ventral tegmental area (VTA) that release dopamine (DA) in the nucleus accumbens (NAcc) and prefrontal cortex (PFC), among other brain areas (Berthoud et al., 2017). Regarding the reward for palatable foods, it has been shown that in animals fed with control food, the palatable food exposition, such as cheetos® and kinder® chocolate, or to sucrose-sweetened solutions, produces an increase in NAcc DA extracellular levels (Bassareo and Di Chiara, 1997; 1999; Hajnal et al., 2004). In obesity, palatable foods deregulate dopaminergic signaling in the striatum (Wallace and Fordahl, 2022), showing that obese have lower availability of striatal DA receptor type 2 (D_2_), an essential protein for controlling compulsive behaviors (Wang et al., 2001). In this context, we have published that chronic exposure to high fat diet (HFD) from postnatal day (PND) 21–62 (6 weeks) (decreases the functioning of the DA transporter (DAT) and the tonic DA release in NAcc (Plaza-Briceno et al., 2023). LS neurons also regulate VTA dopaminergic neurons (Sotomayor et al., 2005; Vega-Quiroga et al., 2018), participating as a relay station between homeostatic and hedonic brain areas.

The CRFergic system comprises the neuropeptides corticotropin-releasing factor (CRF) and urocortins (UCN 1, 2, and 3), as well as their receptors (CRF_1_ and CRF_2_) and the CRF-binding protein (CRF-BP) (Vale et al., 1981; Behan et al., 1995). The functional role of CRF receptors in extra-hypothalamic regions such as the LS has been studied under stressful conditions, including food deprivation, predator odor exposure, social subordination stress and chronic drug administration (Martin and Timofeeva, 2010; Mitra et al., 2011; Sotomayor-Zarate et al., 2013; Mirrione et al., 2014). In this context, LS UCN infusions decrease food intake, an effect blocked by the CRF_2_ antagonist Astressin-2B (Bakshi et al., 2007). Conversely, the activation of LS neurons induced by acute restraint stress contributes to the hypophagic response observed only in males, whereas chronic exposure to a HFD abolishes stress-induced hypophagia and the LS neuronal activation (Bales et al., 2022). However, it remains unknown whether chronic HFD exposure directly affects the CRFergic system within the LS. Therefore, this study evaluated behavioral, neurochemical, and molecular changes in LS CRFergic and dopaminergic neurotransmission in rats exposed to a HFD for 6 weeks.

## 2 Materials and methods

### 2.1 Reagents

DA, DOPAC, NA, 5-HT, 5-HIAA, glutamate, and GABA standards, EDTA, and 1-octanesulfonic acid were purchased from Sigma-Aldrich, Inc. (St. Louis, Missouri, United States of America). All other reagents were of analytical and molecular grades.

### 2.2 Animals

#### 2.2.1 Animal welfare

**FIGURE 1:**
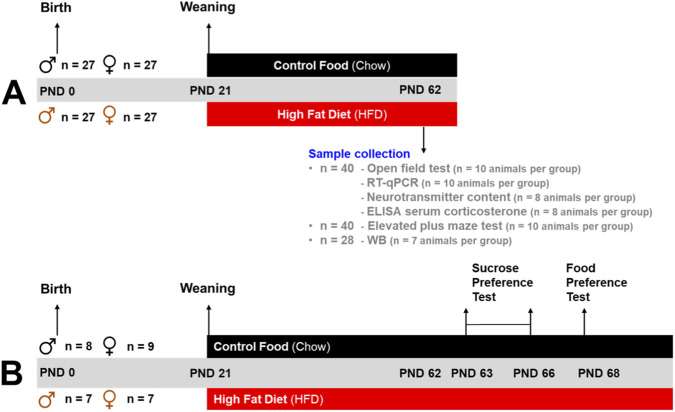
Experimental timeline **(A)**. Males and females were divided into two groups: control groups were fed with a chow diet, and High fat diet (HFD) groups were fed with HFD from postnatal day (PND) 21 (weaning) to PND 62. At PND 62, animals were euthanized. The brain was removed, and the LS was microdissected for posterior Wester Blott (WB), real-time qPCR (RT-qPCR), ELISA, and neurotransmitter tissue content analysis and behavioral tests (OF and EPM). Experimental timeline **(B)**. Males and females were divided in control and High fat diet (HFD) groups and exposed to sucrose preference test (PND 63–66) and food preference test (PND 68).

One hundred and thirty-nine Sprague-Dawley rats from different litters were used in this study. Forty animals were used in the open field test, their microdissected brain tissues were analyzed by RT-qPCR, neurotransmitter tissue content and recollected blood for the analysis of serum by ELISA. Another 40 animals were used in the elevated plus maze test, 28 animals for western blots and 31 animals for an extended protocol to evaluate sucrose and food preference tests. The animals were housed per experimental groups, initially 5 rats per cage from weaning (day 21) until PND 40 and after 3 rats per cage in an animal holding room at the Instituto de Fisiología (Universidad de Valparaíso), under daily supervision until experiment completion (PND 62 or PND 68) (Figure 1). The holding room was maintained at a controlled temperature (22 °C ± 2 °C) and humidity (55% ± 5%), with a 12:12 h light-dark cycle, with lights on at 08:00 h. Tap water and chow diet (isopro® RMH 3000, LabDiets, St. Louis, Missouri, United States of America) with a composition of 14% fat, 26% protein, and 60% carbohydrate, 4.1 kcal/g, were provided *ad libitum* for control animals. Drinking water was supplemented with 5% (^w^/_v_) analytical-grade sucrose to simulate chronic consumption of sugary drinks and increase total caloric intake (representative of a Western diet), and HFD (D12492, Research Diets®, New Brunswick, NJ. United States of America) with a composition of 60% fat, 20% protein, and 20% carbohydrate, 5.21 kcal/g, were provided *ad libitum* for HFD animals.

#### 2.2.2 Sample collection

Samples were collected on PND 62, or on PND 68 in animals that underwent the food and sucrose preference tests, under deep isoflurane anesthesia using a small-animal anesthesia system (Model 510, RWD Life Science Co., Ltd., Shenzhen, China). Briefly, animals were placed in an induction chamber (3% isoflurane with air flow at 0.8 L/min) for at least 3 min. Rapid decapitation was performed using a guillotine for smaller animals (model ST2 55–0012, Harvard Apparatus, United States of America).

#### 2.2.3 Tissue microdissection

Upon decapitation, gonadal, retroperitoneal fat, and brown fat were dissected for weighing, as these tissues are susceptible to changes due to obesogenic diets. Brains were rapidly retrieved from the head, and microdissected on ice using a brain matrix for coronal sections (model 68,711, RWD Life Science, Shenzhen, P.R. China), from which the following structures were obtained: LS, NAcc, Striatum, VTA, Amy, Hipp and LH. In addition, the pituitary gland was obtained from the skull base as control tissue. All these brain nuclei and pituitary were weighed on an analytical balance and then stored in dry 1.5 mL Eppendorf tube at −80 °C for further analysis.

### 2.3 Behavioral tests

Behavioral tests were performed in PND 62 with a total of 80 animals. Forty animals were used in the open field test, and 40 in the elevated plus maze test. For each test, 10 animals were used per experimental group. Importantly, microdissected tissues from only the animals used for the open-field test were analyzed by RT-qPCR and serum corticosterone determination.

#### 2.3.1 Open field (OF) test

This test was performed to evaluate anxiety-like behaviors and total locomotor activity during a 30-min period. Along with the above, anxiety-like behaviors during the first 5 min of the test were also analyzed. Briefly, each animal was placed in the center of a black acrylic cage (50 cm long x 50 cm wide x 50 cm high) and illuminated at 300 ± 20 lux (measured with a digital luxmeter, model #LX-110B, Weafo Instrument Co., United States of America). Behavior was recorded for 30 min, and the video was analyzed using ANY-maze software (ANY-maze v.7.4, Stoelting Co., IL, United States of America). The software divides the arena into 16 equal squares, identifying central and peripheral zones. Locomotor activity was measured every 5 min and accumulated over the 30-min period. The number of entries and time spent in both the central and peripheral zones were also analyzed. After the test, the arena was cleaned with a 5% ^v^/_v_ ethanol solution before being reused.

#### 2.3.2 Elevated plus maze (EPM) test

Briefly, rats were placed in the center of a maze (15 × 15 cm) that connected two open arms (60 cm length x 15 cm width) and two closed arms (60 cm length x 15 cm width x 30 cm height). The maze was positioned on a platform 100 cm above the floor, and the illumination for the open and closed arms were 300 ± 10 lux and 210 ± 10 lux, respectively (measured using a digital luxmeter, model #LX-110B, Weafo Instrument Co., United States of America). The behavior of each animal was recorded for 5 min, and the video was analyzed using ANY-maze software (ANY-maze v.7.4, Stoelting Co., IL, United States of America). The number of entries and time spent in the open and closed arms were analyzed. After the test, the apparatus was cleaned with a 5% ^v^/_v_ ethanol solution before being reused.

The sucrose preference test was performed between days 63 and 66, while the food preference test was performed on day 68 after birth (PND) in a total of 31 animals.

#### 2.3.3 Sucrose consumption preference test

The sucrose preference test (Sucrose, Winkler) was performed from PND 63 to PND 66. Four hours before the test, the rats were deprived of water, then for a further 4 hours, each rat was placed in an individual box with access to five test tubes, each with a volume of 100 mL, containing the following concentrations of liquids: water, 2.5% ^w^/_v_ sucrose, 5% ^w^/_v_ sucrose, and 10% ^w^/_v_ sucrose. The time chosen to perform the test was at dusk to record fluid consumption during the rats’ most active time.

Red light was used to illuminate and observe the consumption of liquids from each test tube without disturbing the rats. The consumption of each liquid concentration was recorded immediately. After the last measurement, each rat was taken back to its initial box with access to food and water *ad libitum*. The experiment was conducted using a total of 7 animals per group.

#### 2.3.4 Food preference test

This was carried out on both rat groups fed with obesogenic and control diet, respectively. To assess food consumption in both groups on DPN 67, food was removed from the animals’ cages at 5:00 p.m., and only drinking water was left available. The next day (DPN 68), at 9:00 a.m., the animals were individually transferred to cages with wire floors that had two removable food modules that could be weighed to quantify food consumption (BIODAQ cages w/2 openings, Research Diets, Inc., NJ, United States of America). They were left in these cages for 90 min to allow them to become accustomed to them. Then, at 10:30 a.m., HFD food was added to one module and control food was added to the other module and left for 1 h, during which time the animals were able to choose which food to eat. At the end of the procedure, the animals were returned to their usual cages. The experiment was conducted using a total of 7 animals per group.

### 2.4 Serum corticosterone determination

At PND 62, after application of the open field test, subsequent sample collection, blood was collected from the trunk of rats (n = 8 rats per experimental group) in 1.5 mL tubes. These tubes were centrifuged for 10 min at 4 °C and 10,000 rpm to obtain the serum, which was kept at −80 °C until further analysis. Corticosterone concentration was analyzed using an ELISA Kit based on competitive inhibition (catalog number ELK8633, ELK Biotechnology, Denver, CO, United States of America), following the manufacturer instructions. The kit has intra-assay and inter-assay variations of less than 8% and 10%, respectively. The detection range for corticosterone was 1.57–100 ng/mL, and its sensitivity was 0.5 ng/mL. The plate was measured spectrophotometrically at a wavelength of 450 nm (Epoch™, BioTek Instruments Inc., Winooski, VT, United States of America).

### 2.5 Western blots

The LS was stored at −80 °C to determine CRF protein levels at PND 62 (n = 7 rats per experimental group). Nuclei were homogenized with RIPA buffer (pH = 8.0, 150 mM NaCl, 50 mM Tris-HCl, 1% (^v^/_v_) Nonidet P40, 0.1% (^w^/_v_) sodium dodecyl sulphate (SDS), 2 mM EDTA, 1.5 mM PMSF, and a protease inhibitor cocktail (catalog N° G6521, Protease Inhibitor Cocktail 50X, Promega™ Corporation, Madison, WI, United States of America) using a sonicator (Q55 sonicator, QSonica, Newtown, CT, United States of America). Total protein concentration was determined by the Bio-Rad Protein Assay (Bio-Rad Laboratories, Inc., Richmond, CA, United States of America) using a microplate spectrophotometer (Epoch™, BioTek Instruments Inc., Winooski, VT, United States of America) and protein samples (30 μg) were separated by SDS-PAGE on 8% polyacrylamide gels under denaturing conditions (4% concentrator gel, 10% resolute gel). Proteins were transferred to nitrocellulose membrane (catalog N° 88,018, 0.45 μm pore, Thermo Fisher Scientific Inc., Waltham, MA, United States of America) at 350 mA for 1.5 h. Non-specific membrane binding sites were blocked with 5% skim milk in T-TBS (0.1% Tween-20, 20 mM TBS, 137 mM NaCl) for GAPDH, and 5% BSA in T-TBS was used for CRF, for 1 h at room temperature. Nitrocellulose membranes were incubated with T-TBS overnight at 4 °C and then incubated with the rabbit anti-CRF antibody (catalog N° ab184238, Abcam, Cambridge, MA, United States of America) (1:1.000, overnight incubation) and rabbit anti-GAPDH antibody (catalog N° ab9485, Abcam, Cambridge, MA, United States of America) (1:10.000, 1 h incubation). The antibody complexes were detected using a goat anti-rabbit IgG Fc conjugated with HRP (Catalog N° ab97200, Abcam, Cambridge, United Kingdom). For chemiluminescent detection, the SuperSignal™ Kit (catalog number 34075, Thermo Scientific, Rockford, IL, United States of America) was used, and the images of the membranes were obtained using Omega Lum™ G (Gel Company, San Francisco, CA, United States of America). The images were analyzed using ImageJ™ software (http://rsbweb.nih.gov/ij/).

### 2.6 RT-qPCR

Real-time RT-qPCR was used to determine changes in the mRNA encoding D_1_, D_2_, GABA_A_, GABA_B_, CRF_1_ and CRF_2_, in LS, NAcc, Amy, Pit, Hipp and LH at PND 62. Total RNA was extracted using the E.Z.N.A. Total RNA Kit I (catalog N° R6834-02, Omega Biotek, Inc., Norcross, GA, United States of America) according to the manufacturer’s instructions. RNA was quantified using a microplate spectrophotometer (Epoch™, Biotek Instruments Inc., Winooski, VT, United States of America), and RNA integrity was assessed through agarose gel electrophoresis. Total RNA from each sample was reverse transcribed with PrimeScript RT reagent Kit (catalog N° RR047A, TaKaRa, Bio Inc., CA, United States of America), according to the manufacturer’s instructions. Real-time RT-PCR was performed using TaqMan assays (Life Technologies Corporation, CA, United States of America) for D_1_ (catalog N° Rn03062203_s1, VIC dye-labelled), D_2_ (catalog N° Rn00561126_m1, FAM dye-labelled), GABA_A_ (catalog N° Rn00524612_m1, FAM dye-labelled), GABA_B_ (catalog N° Rn00578911_m1, VIC dye-labelled), CRF_1_ (catalog N° Rn00578611_m1, VIC dye-labelled), CRF_2_ (catalog N° Rn00575617_m1, FAM dye-labelled), and 18s (Catalog N° Cat: Rn03928990_g1, VIC dye-labelled) genes. The cycling conditions were 50 °C for 2 min, followed by 95 °C for 2 min, and then 40 cycles of 95 °C for 3 s and 60 °C for 30 s, using a real-time PCR system (CFX96 Touch Real-Time PCR, Bio-Rad Laboratories Inc., Hercules, CA, United States of America). Results were expressed as fold change by the 2^−ΔΔCT^ (Livak and Schmittgen, 2001).

### 2.7 Tissue content of neurotransmitters and metabolites

On the day of analysis, each brain tissue sample (NAcc, VTA and striatum) previously stored at −80 °C was placed on ice and 0.4 mL of 0.2 M perchloric acid was added. Subsequently, each sample was homogenized for 20 s using a sonicator (Q55 sonicator, QSonica, Newtown, CT, United States of America) on ice. The homogenate was centrifuged at 12,000 g for 15 min at 4 °C (model Z233MK-2, Hermle Labor Technik GmbH, Wehingen, Germany), and the supernatant was filtered using HPLC syringe filters (model EW-32816-26; Cole-Parmer, Vernon Hills, IL, United States of America).

#### 2.7.1 DA quantification

Ten microliters of final clear supernatant were injected into HPLC coupled to electrochemical detection (ED) with the following configuration: Isocratic pump (model PU-2080 Plus, Jasco Co. Ltd., Tokyo, Japan), C18-column (model Kromasil 100-3.5-C18, AkzoNobel, Bohus, Sweden), and electrochemical detector (model LC-4C, Bioanalytical System Inc., West Lafayette, IN, United States of America) set at 0.700 V (oxidation potential), 0.5 nA (sensitivity), and 0.03 Hz (electrical noise). The composition of the mobile phase was 0.1 M NaH_2_PO_4_, 1.5 mM 1-octanesulfonic acid, 1.28 mM EDTA, 2.0% (^v^/_v_) tetrahydrofuran, and 4.5% (^v^/_v_) CH_3_CN (pH 3.6). It was pumped at a flow rate of 0.15 mL/min, and the retention times for NA, DOPAC, DA, 5-HIAA and 5-HT were 6.8, 9.8, 12.4, 15.2, 17.7, and 29.0 min, respectively. The quantification was performed using a calibration curve for DA (Program ChromPass, Jasco Co. Ltd., Tokyo, Japan). The concentration was expressed as pg/mg of wet tissue.

#### 2.7.2 Glutamate and GABA quantification

Twenty microliters of final clear supernatant were mixed with 4 μL of borate buffer (pH 10.8) and 4 μL of fluorogenic reagent (20 mg of orthophthaldehyde and 10 μL of β-mercaptoethanol in 5 mL of absolute ethanol). The mixture was shaken for 90 s to complete the derivatization and injected into HPLC coupled to fluorometric detection (FD) with the following configuration: Isocratic pump (model PU-4180, Jasco Co. Ltd., Tokyo, Japan), C18-column (model Kromasil 100-3.5-C18, AkzoNobel, Bohus, Sweden), and fluorescence detector (model FP-4025, Jasco Co. Ltd., Tokyo, Japan). The composition of the mobile phase was 0.1 M NaH_2_PO_4_ and 24.0% (^v^/_v_) CH_3_CN (pH 5.7). It was pumped at a flow rate of 0.8 mL/min, and the retention times for Glutamate and GABA were 1.5 and 13.0 min, respectively. The quantification was performed using a calibration curve for Glutamate and GABA (Program ChromNAV 2.0, Jasco Co., Ltd., Tokyo, Japan). The concentration was expressed as ng per mg of wet tissue.

### 2.8 Statistical analysis

Results are expressed as mean ± SEM. Results were analyzed using 2-way ANOVA, followed by Fisher’s LSD multiple comparisons test. Statistical analyses were performed using GraphPad Prism version 10.5.0 (GraphPad Software, San Diego, CA, United States of America), and a P-value of less than 0.05 was considered statistically significant.

## 3 Results

### 3.1 Effect of chronic exposure to HFD on murinometric parameters and serum corticosterone levels

**FIGURE 2:**
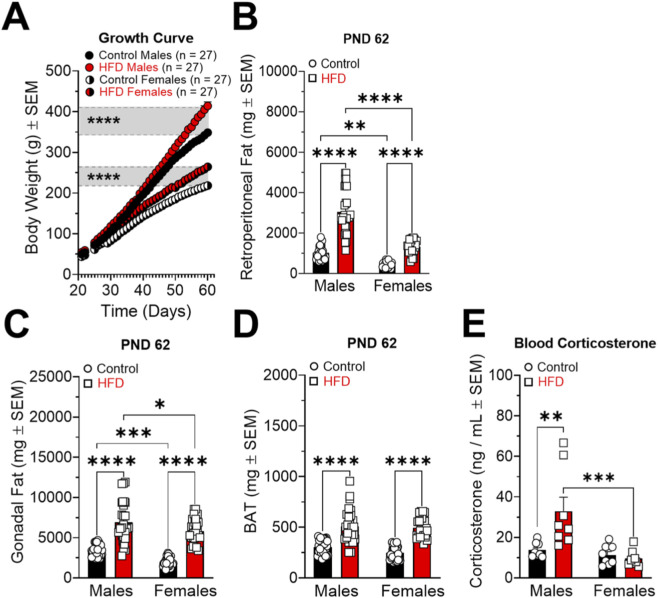
Effects of a high fat diet (HFD) for 6 weeks on male and female body weight (**(A)**, g ± SEM), retroperitoneal fat (**(B)**, mg ± SEM), gonadal fat (**(C)**, mg ± SEM), brown adipose tissue (BAT) (**(D)**, mg ± SEM) and corticosterone levels (**(E)**, ng/mL ± SEM). *P < 0.05, **P < 0.01, ***P < 0.001 and ****P < 0.0001.

As illustrated in Figure 2A, exposure to a HFD resulted in a significant increase in body weight in both male and female rats ([males: Interaction F_(37,1976)_ = 3.581; P < 0.0001. Time F_(37,1976)_ = 478.7; P < 0.0001. Diet F_(1,1976)_ = 220.4; P < 0.0001] [females: Interaction F_(37,1976)_ = 3.755; P < 0.0001. Time F_(37,1976)_ = 472.2; P < 0.0001. Diet F_(1,1976)_ = 869.2; P < 0.0001]), as well as in retroperitoneal (Figure 2B; [Interaction F_(1,104)_ = 24.53; P < 0.0001. Sex F_(1,104)_ = 94.05; P < 0.0001. Diet F_(1,104)_ = 153.4; P < 0.0001]), gonadal (Figure 2C; [Interaction F_(1,104)_ = 0.5065; P = 0.4782. Sex F_(1,104)_ = 18.64; P < 0.0001. Diet F_(1,104)_ = 148.8; P < 0.0001]), and brown adipose tissue mass (Figure 2D; [Interaction F_(1,104)_ = 1.022; P = 0.3143. Sex F_(1,104)_ = 2.782; P = 0.0983. Diet F_(1,104)_ = 117.7; P < 0.0001]), compared to animals maintained on a control diet. Additionally, these males displayed significantly elevated serum corticosterone levels compared with both control males and HFD-consuming females (Figure 2E; [Interaction F_(1,28)_ = 7.959; P = 0.0087. Sex F_(1,28)_ = 12.29; P < 0.0001. Diet F_(1,28)_ = 5.555; P = 0.0257]). This response was not observed in females, suggesting a sex-specific physiological adaptation to the HFD. The analysis comprised 27 animals per group for body weight and adipose tissue and 8 animals per group for serum corticosterone levels.

As illustrated in Supplementary Figure S1, HFD consumption caused significant alterations in the adipose tissue of the 31 animals included in the food and sucrose test. Animals exposed to HFD resulted in a significant increase in the mass of retroperitoneal (1A; Interaction F_(1,21)_ = 21.78; P < 0.0001. Sex F_(1,21)_ = 69.15; P < 0.0001. Diet F_(1,27)_ = 144.4; P < 0.0001), gonadal (1B; Interaction F_(1,27)_ = 1.603; P = 0.2163. Sex F_(1,27)_ = 69.82; P < 0.0001. Diet F_(1,27)_ = 149.5; P < 0.0001), and brown (Figure 1C; Interaction F_(1,27)_ = 2.193; P = 0.1503. Sex F_(1,27)_ = 23.27; P < 0.0001. Diet F_(1,27)_ = 71.70; P < 0.0001) adipose tissues, compared to animals maintained on a control diet.

### 3.2 Effect of chronic exposure to HFD on locomotor activity and anxiety-like behaviors

**FIGURE 3:**
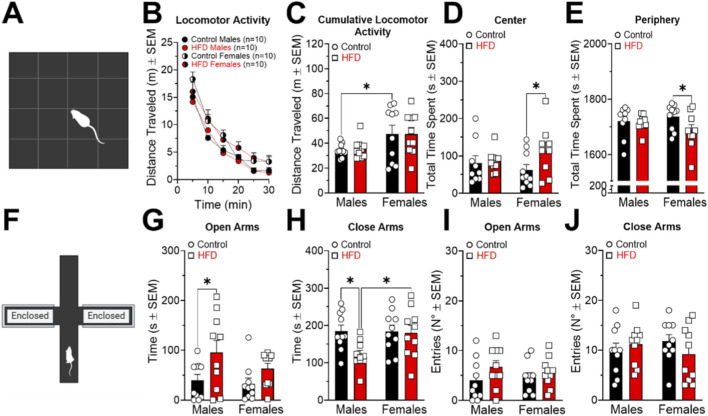
Effects of a high fat diet (HFD) on behavior in males and females in open field (OF) test **(A)** and elevated plus maze (EPM) test **(F)**. Locomotor activity (**(B)**, m ± SEM), cumulative locomotor activity in 30 min (**(C)**, m ± SEM), total time spent in the center (**(D)**, s ± SEM), total time spent in the periphery (**(E)**, s ± SEM), time spent in the open arms (**(G)**, s ± SEM), time spent in the close arms (**(H)**, s ± SEM), entries in the open arms (**(I)**, N° ± SEM), entries in the close arms (**(J)**, N° ± SEM) were assessed. *P < 0.05, **P < 0.01,****P < 0.0001.

Two behavioral tests were performed: the OF (Figures 3A–E) and the EPM tests (Figures 3F–J). In the OF test, no significant differences in general locomotor activity were observed across any of the experimental groups (Figure 3B). However, analysis of cumulative locomotor activity revealed significant differences in animals fed with the control diet (Figure 3C; Interaction F_(1,36)_ = 0.02018; P = 0.8878. Sex F_(1,36)_ = 7.471; P = 0.0097. Diet F_(1,36)_ = 0.01761; P = 0.8952). Significant differences were observed in females in both the total time spent in the central area (Figure 3D; Interaction F_(1,34)_ = 1.955; P = 0.1711. Sex F_(1,34)_ = 0.08601; P = 0.7711. Diet F_(1,34)_ = 2.918; P = 0.0967) and the peripheral area of the arena (Figure 3E; Interaction F_(1,34)_ = 1.985; P = 0.1679. Sex F_(1,34)_ = 0.09367; P = 0.7614. Diet F_(1,34)_ = 2.953; P = 0.0948), specifically in animals that consumed the HFD.

In the EPM test (Figures 3F–J), significant differences were detected in the seconds of time spent in the open arms (Figure 3G; Interaction F_(1,36)_ = 0.6529; P = 0.4244. Sex F_(1,36)_ = 1.628; P = 0.2101. Diet F_(1,36)_ = 7.765; P = 0.0084) and closed arms between control males and those fed with HFD (Figure 3H; Interaction F_(1,36)_ = 3.092; P = 0.0872. Sex F_(1,36)_ = 2.857; P = 0.0996. Diet F_(1,36)_ = 4.044; P = 0.0519). No significant differences were observed in the number of entries into either the open (Figure 3I) or closed arms (Figure 3J) between the HFD and control diet groups.

### 3.3 Effect of chronic exposure to HFD on LS CRF levels

**FIGURE 4:**
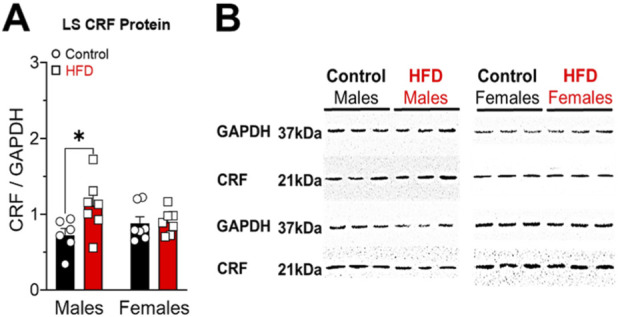
CRF protein levels were measured in the lateral septum (LS) of male and female rats fed either a control diet or a high fat diet (HFD) **(A)**. Data are presented as mean ± SEM of arbitrary units of CRF immunoreactivity normalized to GAPDH. Representative immunoblots for CRF and GAPDH are shown on the left. Single bands were detected at ∼21 kDa for CRF and ∼37 kDa for GAPDH **(B)**. A significant increase in CRF signal intensity was observed in HFD males compared with their respective controls (P < 0.05).

As illustrate in Figure 4 there is an elevated level of CRF in the LS of male rats fed with HFD, compared to control males (Figure 4A; Interaction: F_(1,23)_ = 3.614; P = 0.0699. Sex: F_(1,23)_ = 0.1064; P = 0.7472. Diet: F_(1,23)_ = 4.412; P = 0.0468). No significant differences were observed in the female subjects. Representative immunoblots for CRF and GAPDH in male and female samples are shown on the left. An increase in CRF signal intensity was observed in male HFD rats relative to controls (Figure 4B).

### 3.4 Effect of chronic exposure to HFD on the expression of D_1_, D_2_, GABA_A_, GABA_B_, CRF_1_, and CRF_2_ genes in the LS and NAcc pathway

**FIGURE 5:**
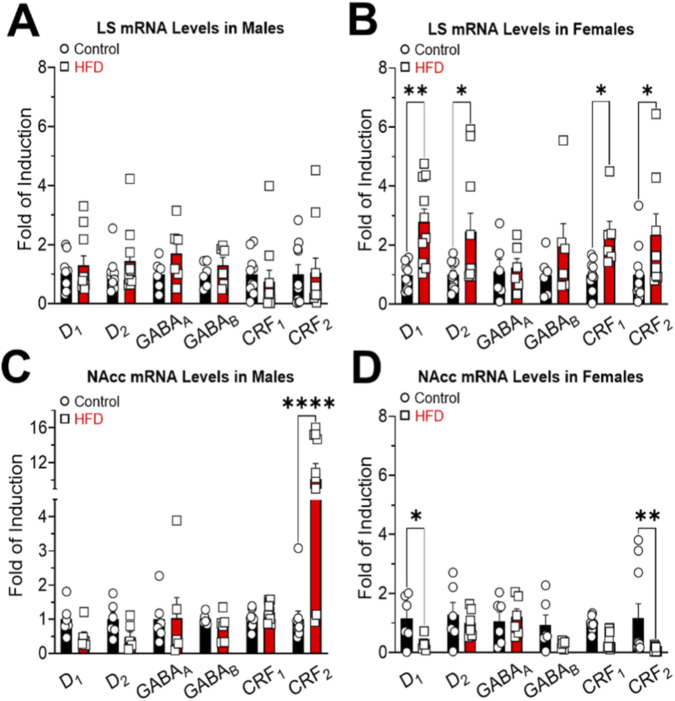
D_1_, D_2_, CRF_1_, and CRF_2_ mRNA expression in the lateral septum (LS) and nucleus accumbens (NAcc) of male **(A,C)** and female **(B,D)** control and HFD rats. All data have been normalized for 18S expression levels within the same sample. Results are expressed as fold induction relative to the control group and represent mean ± SEM. *P < 0.05 and **P < 0.01.

In LS, no significant differences in receptor gene expression were detected in males (Figure 5A), with no main effects or interactions (Interaction: F_(5,92)_ = 0.4911; P = 0.7821. Gene: F_(5,92)_ = 0.4946; P = 0.7795. Diet: F_(1,92)_ = 1.733; P = 0.1913). On the other hand, significant differences in the gene expression of D_1_, D_2_, CRF_1_, and CRF_2_ receptors were exclusively observed in HFD-fed females compared to controls, showing higher mRNA expression of these receptors in animals fed an HFD (Figure 5B). Statistical analysis revealed a significant main effect of gene (F_(3,48)_ = 14.25; P < 0.0001), but not of diet (F_(1,48)_ = 1.291; P = 0.2615) or interaction (F_(3,48)_ = 1.569; P = 0.2091).

In the NAcc of males, only CRF_2_ expression was significantly altered, with higher levels in HFD-fed animals compared to controls (Figure 5C). However, HFD-fed females exhibited a significantly reduced expression of D_1_ and CRF_2_ in comparison to controls (Figure 5D). Statistical analysis revealed the main effect of gene (F_(3,46)_ = 35.11; P < 0.0001), but not of diet (F_(1,46)_ = 2.157; P = 0.1487) or interaction (F_(3,46)_ = 1.315; P = 0.2809). However, no significant main effects or interactions were found (Interaction: F_(3,50)_ = 1.232; P = 0.3079. Gene: F_(3,50)_ = 1.228; P = 0.3092. Diet: F_(1,50)_ = 0.1057; P = 0.7464).

### 3.5 Effect of chronic exposure to HFD on the expression of CRF_1_ and CRF_2_ genes in brain areas associated with CRFergic activity, such as amy, pit, LH and hipp

**FIGURE 6:**
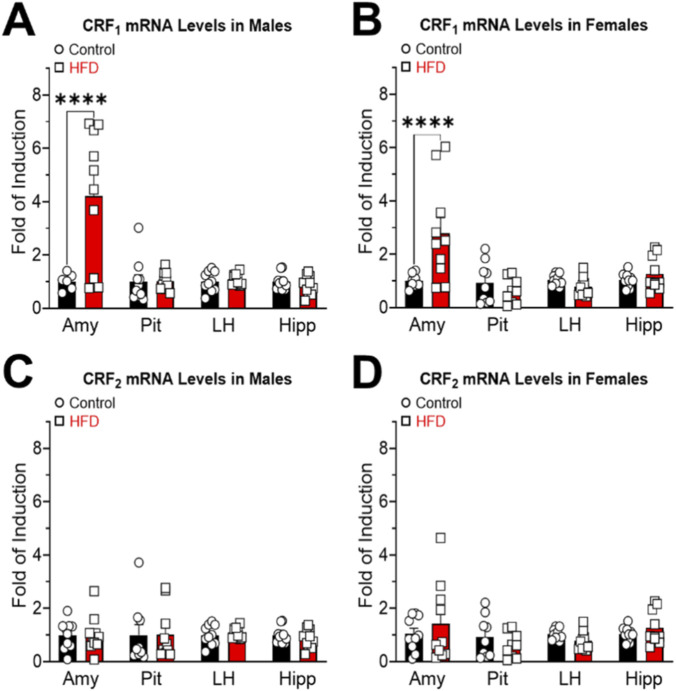
CRF_1_ and CRF_2_ mRNA expression in amygdala (Amy), pituitary (Pit), lateral hypothalamus (LH) and hippocampus (Hipp) of male **(A,C)** and female **(B,D)** control and HFD rats. All data have been normalized for 18S expression levels within the same sample. Results are expressed as fold induction relative to the control group and represent mean ± SEM. ****P < 0.0001.

The analysis of CRF_1_ receptor gene expression revealed a significant difference exclusively in the Amy of both HFD-fed males (Figure 6A; Interaction: F_(3,68)_ = 10.43; P < 0.0001. Nucleus: F_(3,68)_ = 10.41; P < 0.0001. Diet: F_(1,68)_ = 11.56; P = 0.001) and females (Figure 6B; Interaction: F_(3,68)_ = 6.697; P = 0.0005. Nucleus: F_(3,68)_ = 7.055; P = 0.0003. Diet: F_(1,68)_ = 3.906; P = 0.0522) compared to animals on the control diet. No significant differences were observed in CRF_1_ mRNA expression in the Pit, LH, or Hipp.

No differences were identified in the expression of the CRF_2_ receptor gene, neither in any area evaluated, nor in either of the sexes evaluated (Figure 6C; Interaction: F_(3,71)_ = 0.1099; P = 0.9541; Nucleus: F_(3,71)_ = 0.1080; P = 0.9551; Diet: F_(1,71)_ = 0.0032; P = 0.9551; Figure 6D; Interaction: F_(3,70)_ = 1.123; P = 0.3458; Nucleus: F_(3,70)_ = 1.723; P = 0.1702; Diet: F_(1,70)_ = 0.0052; P = 0.9425).

### 3.6 Effect of chronic exposure to HFD on tissue content of DA, DOPAC, NA, 5-HT, 5-HIAA, GLU, and GABA in VTA, NAcc, and striatum

**FIGURE 7:**
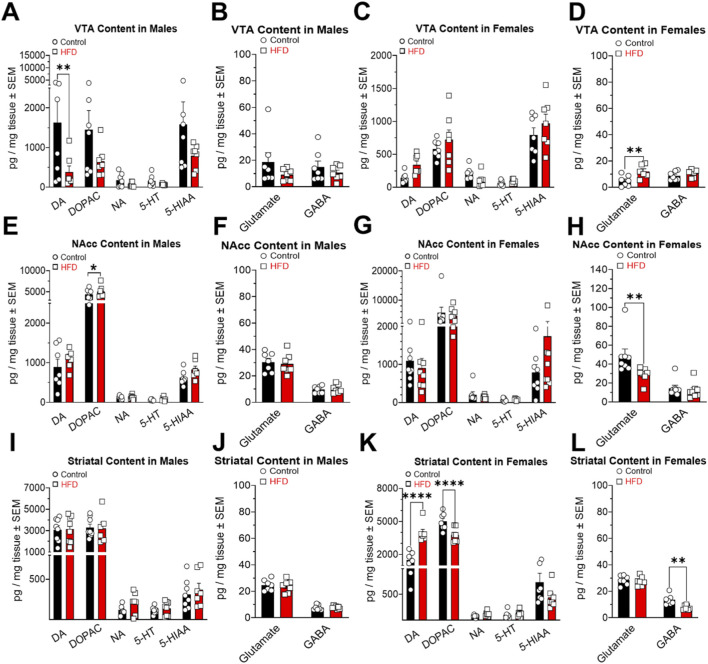
Effects of a high-fat diet (HFD) on tissue levels of dopamine (DA), DOPAC, noradrenaline (NA), 5-HT, 5-HIAA, GABA and Glutamate in VTA **(A–D)**, NAcc **(E–H)** and striatum **(I–L)** in male and female rats. Data (mean ± SEM) are expressed in pg/mg tissue. *P < 0.05, **P < 0.01 and ****P < 0.0001.

Neurochemical analysis of the VTA revealed significant differences in DA tissue content with HFD-fed animals exhibiting lowers concentrations than controls (Figure 7A; Interaction: F_(4,60)_ = 1.243; P = 0.3023. Content: F_(4,60)_ = 5.743; P = 0.0006. Diet: F_(1,60)_ = 9.340; P = 0.0033). In females, significant differences were observed only in glutamate levels, with HFD-fed animals exhibiting higher concentrations than controls (Figure 7D; Interaction: F_(1,22)_ = 3.082; P = 0.0931. Content: F_(1,22)_ = 0.5460; P = 0.4678. Diet: F_(1,22)_ = 8.542; P = 0.0079). No significant differences were observed in glutamate or GABA content in males (Figure 7B), nor in DA, DOPAC, NA, 5-HT, or 5-HIAA levels in females (Figure 7C).

A substantial increase in the DOPAC content of the NAcc was observed in HFD-fed males compared to controls (Figure 7E; Interaction: F_(4,60)_ = 0.5732; P = 0.6831. Content: F_(4,60)_ = 137.6; P < 0.0001. Diet: F_(1,60)_ = 1.993; P = 0.1632). In females, control animals showed significantly higher glutamate levels than HFD-fed females (Figure 7H; Interaction: F_(1,27)_ = 3.681; P = 0.0657. Content: F_(1,27)_ = 31.87; P < 0.0001. Diet: F_(1,27)_ = 5.993; P = 0.0211). No significant differences were observed in glutamate or GABA content in males (Figure 7F), nor in DA, DOPAC, NA, 5-HT, or 5-HIAA levels in females (Figure 7G).

In the striatum, significant differences were only observed in females. HFD-fed females exhibited elevated DA levels, while control females had elevated levels of DOPAC (Figure 7K; Interaction: F_(4,59)_ = 26.40; P < 0.0001. Content: F_(4,59)_ = 202.3; P < 0.0001. Diet: F_(1,59)_ = 2.774; P = 0.1011) and GABA (Figure 7L; Interaction: F_(1,24)_ = 4.255; P = 0.0501. Content: F_(1,24)_ = 209.1; P < 0.0001. Diet: F_(1,24)_ = 7.720; P = 0.0104). No significant differences were observed in glutamate or GABA content (Figure 7I), nor in DA, DOPAC, NA, 5-HT, or 5-HIAA levels in males (Figure 7J).

### 3.7 Effect of chronic exposure to a high fat diet (HFD) on preference for sucrose solutions in obese and control rats of both sexes, using the free-choice paradigm between bottles with and without sucrose (2.5% ^w^/_v_, 5.0% ^w^/_v_, and 10% ^w^/_v_) between PND 63-66

**FIGURE 8:**
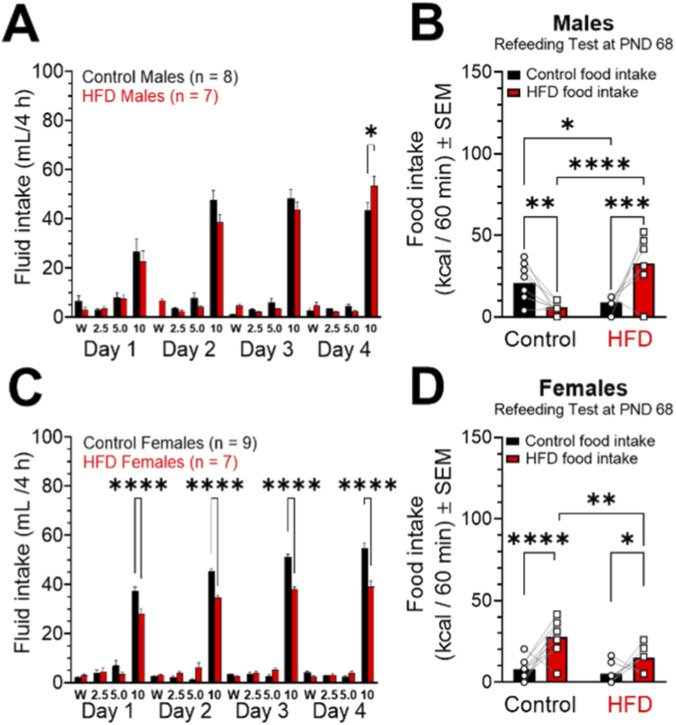
Effects of a high fat diet (HFD) on preference for sucrose solution and food preference (HFD vs. Chow) consumption in males and females. Fluid intake was assessed in males (**(A)**, mL/4 h) and females (**(C)**, mL/4 h). Diet consumption was evaluated in males (**(B)**, kcal/60 min), females (**(D)**, kcal/60 min). *P < 0.05, **P < 0.01, ***P < 0.001, and ****P < 0.0001.

Between days PND 63 and 66, the volume of fluid consumed individually by each rat was recorded after simultaneous exposure to four available solutions *ad libitum*, presented in modified test tubes: water, 2.5% ^**w**^ **/**_**v**_ sucrose, 5% ^**w**^ **/**_**v**_ sucrose, and 10% ^**w**^ **/**_**v**_ sucrose (Figures 8A,C).

Regarding the consumption recorded in males fed HFD and their controls (Figure 8A; Interaction: F_(15,208)_ = 2.179; P = 0.0080; Sex: F_(15,208)_ = 120.3; P < 0.001; Diet: F_(1,208)_ = 0.5752; P = 0.4491), a progressive increase in the intake of the 10% sucrose solution was observed in both groups, with this preference being slightly higher in the control animals on days 1, 2, and 3 of the test. On the fourth day of the test (PND 66), the males in the HFD group consumed significantly more 10% sucrose solution compared to their controls.

In females, both those fed a standard diet and those consuming HFD showed a clear preference for the higher concentration solution (10% ^**w**^ **/**_**v**_ sucrose). However, control females showed significantly higher consumption of this solution compared to HFD females from the first to the fourth day (Figure 8C; Interaction: F_(15,224)_ = 13.82; P < 0.0001; Sex: F_(15,224)_ = 432.4; P < 0.0001; Diet: F_(1,224)_ = 38.67; P < 0.0001).

### 3.8 Effect of chronic exposure to a high fat diet (HFD) on food preference (HFD vs. chow) in obese and control rats of both sexes, using the BioDAQ food measurement system, which is designed to measure accurately and prevent food spillage at PND 68

On day 68, preference for the HFD or Chow diet was determined. This preference was measured for 1 h in kilocalories (Figures 8B,D). The results obtained show that males exposed to the HFD diet showed a higher intake of the high-calorie diet compared to the standard diet and the intake of males in the control group. Consistently, HFD males also showed significantly higher caloric intake. Differentially, control males consumed more chow than HFD males (Figure 8B; Interaction: F_(1,26)_ = 25.68; P < 0.0001; Sex: F_(1,26)_ = 3.607; P = 0.0687; Diet: F_(1,26)_ = 1.303; P = 0.2640).

Regarding food intake in females, females fed the control diet were observed to consume a greater amount of the HFD diet compared to females fed the HFD diet. A similar pattern was observed in caloric intake: control females consumed a significantly greater number of kilocalories derived from the HFD diet compared to HFD females, showing a higher consumption of HFD than control food (Figure 8D; Interaction: F_(1,28)_ = 2.853; P = 0.1023; Sex: F_(1,28)_ = 7.188; P = 0.0122; Diet: F_(1,28)_ = 24.46; P < 0.0001).

## 4 Discussion

It has been demonstrated that during adolescence there is a maturational imbalance between subcortical regions involved in reward processing and cortical areas responsible for executive control (Mills et al., 2014). This principle is relevant for interpreting how early exposure to stressors, such as HFD consumption, may interact with these critical developmental windows and alter the organization of brain regions and circuits involved in emotional and feeding regulation (Zeltser, 2018), including the LS, where direct or indirect dysregulation could increase vulnerability to highly reinforcing stimuli such as palatable food consumption.

In this study, chronic exposure to a high fat diet (HFD) for 6 weeks induced sex-dependent alterations in behavior, neurochemistry, and metabolic parameters, supporting the role of the LS as an integrative node for stress and reward signaling modulated by diet. Both male and female rats fed an HFD showed significant increases in body weight and in retroperitoneal, gonadal, and brown adipose tissues compared with their respective control groups (Figures 2A–D; Supplementary Figure S1), indicating that fat accumulation contributes to weight gain in both sexes. Notably, males exhibited a greater magnitude of adipose tissue expansion and significantly elevated serum corticosterone levels (Figure 2E), whereas females did not show this endocrine response, suggesting sex-dependent physiological adaptations to HFD exposure (Santosa et al., 2017; Wawrzkiewicz-Jalowiecka et al., 2021).

Although the literature has shown that females consuming an HFD often display a higher increase in body weight compared to males, this gain is not necessarily associated with greater adipose tissue accumulation. Females may present lower weight gain efficiency, indicating that despite increased body weight, their bodies convert a smaller proportion of ingested energy into fat mass, a process possibly modulated by hormonal factors. This metabolic feature may confer partial protection against the adverse metabolic effects of HFD (Shi et al., 2022). In addition, we observed a clear sexual dimorphism in the motivational drive toward energy-dense foods (Figures 8B,D). In males, chronic HFD exposure enhanced preference for palatable food, whereas in females, chronic exposure appeared to attenuate the acute response to palatability (Paruthiyil et al., 2018) an effect not observed in control females, who showed a stronger novelty-driven preference for the high-calorie diet.

HFD males spent more time in the open arms of the EPM (Figures 3G,H), a behavioral pattern commonly interpreted as reduced anxiety-like behavior, despite exhibiting elevated serum corticosterone levels. This apparent dissociation between endocrine activation and behavioral output may reflect altered stress processing, as has been reported in models of HPA axis dysregulation (Hewagalamulage et al., 2016). In contrast, HFD females spent more time in the center of the OF (Figure 3D), also consistent with reduced anxiety-like behavior. Unlike males, females did not show significant changes in corticosterone, which may indicate sex-specific differences in the neurobiological response to metabolic challenge rather than a clearly defined compensatory mechanism.

The CRF system mediates stress responses through activation and feedback regulation of the HPA axis, also acting as a central modulator. CRF activity has been shown to play a dimorphic role in the regulation of energy metabolism in animals (Paruthiyil et al., 2018). In this investigation HFD males exhibited increased CRF in the LS (Figure 4), consistent with projections from CRFergic nuclei that connect to this LS (Sheehan et al., 2004). HFD females showed increased CRF_2_ in the LS (Figure 5B), functioning as a compensatory mechanism that modulates the propagation of stress and reward signals (Bakshi et al., 2007). In the NAcc, HFD males displayed elevated CRF_2_ (Figure 5C), associated with increased DOPAC levels and dopamine turnover (Figure 7E), which facilitates dopamine release and hedonic motivation toward HFD and sucrose solutions (Figure 8B) (Baumgartner et al., 2021; Plaza-Briceno et al., 2023). Conversely, HFD females exhibited reduced CRF_2_ in the NAcc (Figure 5D), correlating with lower intake of sucrose and HFD (Supplementary Figure S1C; Figures 8C,D), limiting hedonic activation (Sanson et al., 2025). In the Amy, both sexes presented increased CRF_1_ expression (Figures 6A,B), with elevated corticosterone in males (Figure 2E) contributing to HPA axis activation without necessarily enhancing anxiety-related behavior (Bakshi et al., 2007; Iemolo et al., 2013), whereas in females, CRF_1_ upregulation did not elevate corticosterone, indicating more efficient HPA regulation, likely mediated by CRF_2_ in the LS and NAcc and by gonadal steroids.

It has been demonstrated that dopaminergic pathways control motivated behavior, including that related to feeding. The mesolimbic VTA-NAcc pathway influences motivated behavior by enhancing the willingness to work for rewards (Wallace and Fordahl, 2022). Regarding the dopaminergic profile, males on HFD showed a reduction in DA in the VTA (Figure 7A), which could reflect a compensatory mechanism in response to HFD consumption. However, it has been shown that HFD consumption decreases the expression of tyrosine hydroxylase mRNA and peptide (Li et al., 2009; Sharma and Fulton, 2013), which could be interpreted as a low availability of this neurotransmitter, a phenomenon caused by this type of diet. In the NAcc, an increase in DOPAC was observed (Figure 7E), which could be interpreted as an increase in phasic DA release. The literature has shown that this occurs in response to pleasurable stimuli such as HFD, reflecting hyperactivity of the reward circuit and increased dopaminergic turnover, consistent with evidence from feeding and sucrose preference tests in males consuming HFD (Figure 8) (Cook et al., 2017; Volkow et al., 2017; Plaza-Briceno et al., 2023), therefore, altered dopamine neurotransmission could disrupt satiety circuits between NAcc DA terminals and projections to the hypothalamus. HFD females, in contrast, showed increased striatal DA with reduced DOPAC and GABA in the NAcc (Figures 7K,L), which favors locomotion and exploration without promoting hyperphagia, reflecting a balance between hedonic motivation and intake control (Szczypka et al., 2001). The reduction in glutamate in the NAcc of HFD females (Figure 7H) limits hedonic excitation, modulating reward seeking (Christoffel et al., 2021). These findings suggest the presence of sex dependent responses to metabolic challenges.

Previous studies have linked prolonged HFD consumption to anxiety-like behaviors and cognitive impairment. Moreover, HFD has been reported to disrupt hippocampal glucocorticoid signaling (Sivanathan et al., 2015) and to induce changes in FOS expression within hypothalamic nuclei involved in stress regulation (Noronha et al., 2019). Indeed, large-scale transcriptomic and proteomic analyses indicate that only about one-third of mRNAs show a direct correlation with their protein levels, reflecting the complex influence of post-transcriptional and translational regulatory mechanisms (Gry et al., 2009). These findings suggest that the development of obesity in response to HFD is accompanied by dysregulation of stress-related pathways. Therefore modulation of CRF and DA mediated circuits may contribute to the emergence of anxiety-like behaviors, particularly in males, highlighting the importance of sex-specific neurobiological adaptations in metabolic brain dysfunction.

The behavioral results in the sucrose and food preference tests are consistent with the neurochemical changes. Increased sucrose and HFD consumption in males (Figures 8A,B) align with CRFergic and dopaminergic markers hyperactivity, increased DOPAC in NAcc and CRF_2_ upregulation, promoting compulsive hedonic feeding. In females, lower sucrose and HFD intake relative to control females (Figures 8C,D) could correlate with LS and NAcc CRF_2_ mediated regulation, excitatory-inhibitory balance in DA and glutamate, which could limit overeating and favor controlled exploration.

Based on these findings, it was possible to identify sex-specific effects in response to chronic exposure to a HFD, with particularly notable differences observed at the level of the central nervous system. While females exhibited an obese phenotype, this was not accompanied by the central neurophysiological alterations typically associated with obesity or compulsive consumption of palatable foods, as evidenced by the sucrose preference and feeding tests, in contrast to males (Figure 8).

These findings may reflect sex-dependent differences in the neurobiological response to prolonged HFD exposure rather than the existence of a defined neuroprotective mechanism. Although the present results do not allow direct conclusions regarding the role of gonadal hormones, it is plausible that both estrogenic and androgenic signaling contribute comparably to the modulation of stress- and feeding-related pathways.

Both estrogens and androgens have been implicated in the regulation of energy homeostasis, metabolic balance, and stress reactivity (Hirschberg, 2012; Asarian and Geary, 2013; Kelly and Jones, 2013). Therefore, rather than implying a direct regulatory mechanism, we interpret the observed sex differences as reflecting differential hormonal modulation of CRF-related pathways. In this context, the CRF gene promoter contains both an estrogen response element and an androgen response element (Vamvakopoulos and Chrousos, 1993; Bao et al., 2005), suggesting that both hormonal signals may influence CRF transcription. In addition, the genes encoding estrogen receptors alpha (ERα) and beta (ERβ) are located in close proximity to the CRF gene and have been reported to co-regulate its expression in the PVN (Bao et al., 2005), in addition high expression of ERα and ERβ has been demonstrated in the LS (Milner et al., 2010). Also, androgen receptors have also been identified in this region and other limbic forebrain regions (Bingaman et al., 1994), indicating that sexual hormone signaling in this region could be associated with stress and feeding regulation, although a direct functional role cannot be inferred from the present data.

Overall, our results indicate that chronic HFD exposure is associated with molecular and behavioral changes linked to LS function and related neural circuits. These findings highlight sex as a critical biological variable in vulnerability and adaptation to obesogenic diets, while emphasizing that the mechanistic links between hormones, CRF signaling, and metabolic stress remain to be directly tested.

## Data Availability

The data presented in the study are deposited in the zenodo repository, accession number https://doi.org/10.5281/zenodo.19632378.

## Supplementary material

SUPPLEMENTARY FIGURE S1 The effects of a high fat diet (HFD) over 6 weeks were evaluated, as well as preference for sucrose solution (PND 63–66) and food preference consumption (PND68) on males and females. Retroperitoneal fat (A, mg ± SEM), gonadal fat (B, mg ± SEM), brown adipose tissue (BAT) (C, mg ± SEM). *P < 0.05, ***P < 0.001 and ****P < 0.0001.
