# Supplementary material for: Sex-specific alterations in CRF and dopaminergic markers in the lateral septum and mesolimbic system following chronic high-fat diet exposure in rats
Source: Front Pharmacol. 2026 Apr 30;17:1781720. doi: 10.3389/fphar.2026.1781720 (PMC13172626; doi:10.3389/fphar.2026.1781720)
Supplement: Supplementary file 1 [file Image1.pdf]

Supplementary Figure 1

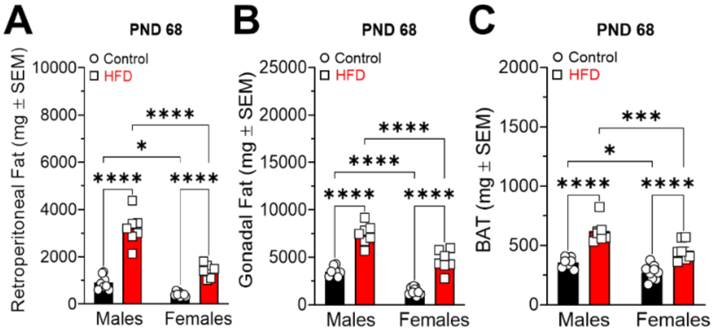

Supplementary figure 1. The effects of a high-fat diet (HFD) over six weeks were evaluated, as well as preference for sucrose solution (PND 63 to 66) and food preference consumption (PND68) on males and females. Retroperitoneal fat (A, mg ± SEM), gonadal fat (B, mg ± SEM), brown adipose tissue (BAT) (C, mg ± SEM). *P < 0.05, ***P < 0.001 and ****P < 0.0001.
